# Epidemiological Analysis, Detection, and Comparison of Space-Time Patterns of Beijing Hand-Foot-Mouth Disease (2008–2012)

**DOI:** 10.1371/journal.pone.0092745

**Published:** 2014-03-24

**Authors:** Jiaojiao Wang, Zhidong Cao, Daniel Dajun Zeng, Quanyi Wang, Xiaoli Wang, Haikun Qian

**Affiliations:** 1 The State Key Laboratory of Management and Control for Complex Systems, Institute of Automation, Chinese Academy of Sciences, Beijing, China; 2 Cloud Computing Center, Chinese Academy of Sciences, Dongguan, China; 3 Institute for Infectious Disease and Endemic Disease Control, Beijing Center for Disease Prevention and Control, Beijing, China; University of Illinois at Chicago, United States of America

## Abstract

**Background:**

Hand, foot, and mouth disease (HFMD) mostly affects the health of infants and preschool children. Many studies of HFMD in different regions have been published. However, the epidemiological characteristics and space-time patterns of individual-level HFMD cases in a major city such as Beijing are unknown. The objective of this study was to investigate epidemiological features and identify high relative risk space-time HFMD clusters at a fine spatial scale.

**Methods:**

Detailed information on age, occupation, pathogen and gender was used to analyze the epidemiological features of HFMD epidemics. Data on individual-level HFMD cases were examined using Local Indicators of Spatial Association (LISA) analysis to identify the spatial autocorrelation of HFMD incidence. Spatial filtering combined with scan statistics methods were used to detect HFMD clusters.

**Results:**

A total of 157,707 HFMD cases (60.25% were male, 39.75% were female) reported in Beijing from 2008 to 2012 included 1465 severe cases and 33 fatal cases. The annual average incidence rate was 164.3 per 100,000 (ranged from 104.2 in 2008 to 231.5 in 2010). Male incidence was higher than female incidence for the 0 to 14-year age group, and 93.88% were nursery children or lived at home. Areas at a higher relative risk were mainly located in the urban-rural transition zones (the percentage of the population at risk ranged from 33.89% in 2011 to 39.58% in 2012) showing High-High positive spatial association for HFMD incidence. The most likely space-time cluster was located in the mid-east part of the Fangshan district, southwest of Beijing.

**Conclusions:**

The spatial-time patterns of Beijing HFMD (2008–2012) showed relatively steady. The population at risk were mainly distributed in the urban-rural transition zones. Epidemiological features of Beijing HFMD were generally consistent with the previous research. The findings generated computational insights useful for disease surveillance, risk assessment and early warning.

## Introduction

Hand, foot, and mouth disease (HFMD) is an infectious disease that mostly affects the health of infants and preschool children. HFMD was first reported in 1958 [1]. It is characterized by a distinct clinical presentation of fever, and vesicular exanthema on the hands, feet, mouth, and buttocks. More than 20 different enteroviruses (EV) have been associated with HFMD [2]–[6]. Vaccine development for the prevention of EV 71-associated HFMD is in progress [7], but HFMD will not be eliminated because many other pathogens (e.g., CoxA 16, strains of EV 71) also cause the disease [8]. Epidemiological surveillance, risk detection, and early warning of HFMD epidemics remain as important issues.

In recent years, HFMD epidemics have been common in the Asia-Pacific region, especially in East and Southeast Asia. Epidemics have occurred in mainland China, Taiwan, Hong Kong [3], [9]–[13], Singapore [14], Japan [15], Malaysia [16], South Korea [17], and Vietnam [2]. HFMD epidemics occurred nationwide in China in 2008 [18]. Since May 2008, the Chinese Ministry of Health has listed HFMD as a notifiable Class-C communicable disease to be included in the surveillance system and reporting network [19].

HFMD epidemics in China vary over space and time. Beijing experienced a serious HFMD epidemic from January 2008 to December 2012 that included 157,707 reported cases. Of these cases, 18,446 occurred in 2008 [13], [20]. The number of cases increased by 32.73% in 2009 (24,483), 146.17% in 2010 (45,408), 67.2% in 2011 (30,842), and 108.87% in 2012 (38,528). Beijing's total population size increased by 3.4% annually during the 2000–2012 period as a result of rapid societal and economic development. The importance of social factors on the epidemiological characteristics of disease are becoming more and more apparent [21]. However, the epidemiological characteristics and space-time patterns of individual-level HFMD cases in a major city such as Beijing, and the interactions between social and natural environments, remain to be determined. The objective of this study was to identify space-time clusters of HFMD cases with high relative risk at a fine spatial scale.

Spatial cluster analysis provides an effective way to identify areas of inadequate health care access and potential environmental and behavioral causal factors [22], [23]. As the risk of HFMD varies over space and time, it is important to identify the regions at risk, the risk factors, the rank of risk, and the exposed population. Using spatial filtering with scan statistics methods provides complementary information and overcomes the weaknesses of using a single method [24], [25], by providing disease risk estimation for areas without HFMD cases and space-time cluster detection of HFMD epidemics. We compared the outcomes from these two methods and explored the spatial patterns of HFMD epidemics. This work provides a foundation for prospective environmental surveillance of the study area.

## Materials and Methods

### Ethics statement

Beijing HFMD data were provided by the Beijing Center for Disease Prevention and Control and were obtained from the National Surveillance System. No informed consent was required because there were no ethical issues relevant to the study design and no individual-level analysis was performed. The information contained in the patient's records was anonymized and de-identified prior to analysis. The data were aggregated and analyzed, and individual participants were not identified.

### Data collection

**Figure 1:**
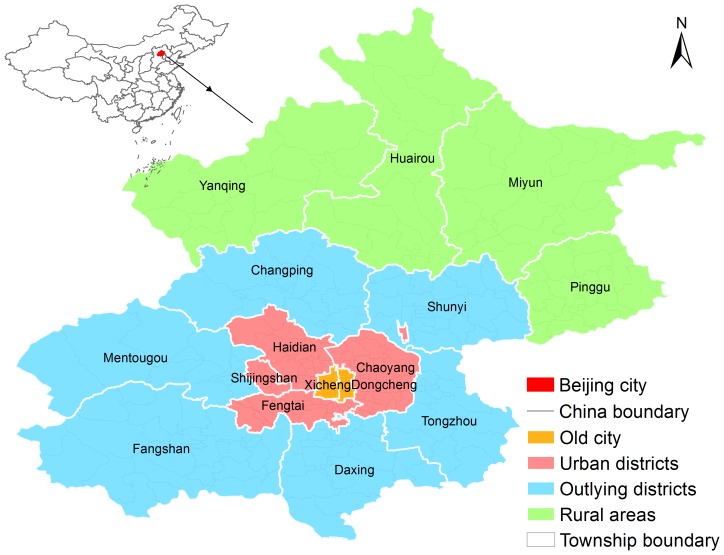
The location of Beijing City in China.

Beijing, the capital of China, is located in northern China at 39°54′50″N 116°23′30″E (Figure 1). Beijing covers a total area of 16,808 km^2^ and consists of 16 districts divided into 309 townships. Data on individual-level HFMD cases from the 2008–2012 period were provided by the Beijing Center for Disease Control and Prevention (BJCDC). The case data included basic social-demographic information and, for some of the cases, pathogen identification. HFMD case records were completed by the physicians treating the patients and were summarized by trained staff. The 2008–2012 demographic data for each census tract were based on 2010 census data and on data published in the Beijing Statistical Yearbook (http://www.bjstats.gov.cn/). The home addresses from the HFMD case records were matched to the geographic coordinates of the township level divisions.

### Epidemiological analysis

The case data were aggregated at the township level. Statistical analyses were performed to describe the epidemiological features (distribution by age, gender, occupation, and pathogen) of HFMD occurrence. Age-gender incidence of HFMD (1/100,000) was defined as the number of HFMD cases in each age-gender group divided by the population size of that age-gender group. Total incidence was defined as the total number of HFMD cases divided by the average population size during the study period. Time series analysis was performed to describe the seasonality distribution of HFMD cases and to detect the peaks in the number of HFMD cases.

### Local Spatial Autocorrelation

Local spatial autocorrelation analysis based on Indicators of Spatial Association (LISA) [26]–[29] was used to measure the spatial autocorrelation of HFMD incidence at the township level for the years of 2008 to 2012. LISA cluster maps were useful to assess the hypothesis of spatial randomness and to detect local hot and cold spots of HFMD epidemics. The univariate LISA could give an indication of the degree of linear relationship (positive or negative) between the values for one value at a given location and the average of neighboring values in the surrounding location. LISA cluster map and LISA significance map were used to visualize the spatial autocorrelation in a data distribution. LISA cluster map suggested two classes of positive spatial correlation (High-High and Low-Low) and two classes of negative spatial correlation (High-Low and Low-High). LISA significance map (a map of P values) highlighted the locations of significant spatial clusters (the default is P<0.05). The spatial weights based on first-order rook contiguity were constructed to identify spatial relationships between the townships in Beijing and GeoDa (V1.4.1) software (Tempe, AZ, USA; https://geodacenter.asu.edu/projects/nij/software) was used to perform the analysis [30].

### Spatial filtering method

Spatial filtering method that was introduced by Rushton and Lolonis [31] was used to estimate disease rates and to detect the areas with significantly high or low disease risk. This method uses non-parametric statistical techniques (i.e., without assumptions about distribution) [32]. It has been used as a spatial interpolation method to study clusters of congenital anomalies, infant mortality, birth defects, and other forms of birth morbidity [24], [31], [33], [34]. In this study, this spatial filtering method was used to map spatial variations in the relative risk of HFMD occurrence and to detect local disease clusters (hot spots and cold spots).

The spatial filtering method consists of a series of steps: (1) Define a fine resolution regular lattice of grid points that cover the study area. (2) Create fixed or adaptive distance circle around each grid point, count the number of cases as the numerator and the population at risk as the denominator within each circle during the time period, and compute the ratio of the two counts for each grid point. (3) Generate the simulated random cases from the population at risk, by assuming that each individual within the population at risk has the same probability of being a case event (Null hypothesis 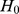). Run the simulation many times (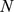). For each round of the simulation, repeat (2) while replacing the real cases with the simulated cases. Thus, an expected distribution of disease rates at each grid point can be constructed, and an empirical P-value of the observed rate can be computed by comparing the observed rate with the expected distribution at this grid point. If the observed rate at a grid point computed from (2) is larger than 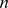 of the 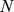 simulated rates, then the P-value of the observed rate is denoted as 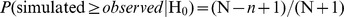 [35].

DMAP IV (Iowa City, IA, USA; www.uiowa.edu/~gishlth/DMAP4/) was used to implement spatial filtering. We first defined the spatial reference grid to cover the study area where the relative risk of HFMD occurrence and test statistics were to be computed. The grid file included 698 points at a resolution of 3 miles. The disease-population file for each year (2008–2012) was then prepared. This file stored township level information (e.g., administrative code, latitude-longitude coordinates, the number of observed cases, the expected number of cases (under 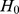), and the population size at risk). A grid rate file was then output by the spatial filtering procedure. The incidence was estimated by dividing the number of observed cases by the population size at risk. Standardized Morbidity Rate (SMR) was estimated as the number of observed cases divided by the expected number of cases (Equations 1, 2, and 3). To estimate relative risk, test statistics such as the Z-value and the P-value were computed. The weighted versions of the output variables were computed using a weighted spatial filter so that the relative risk estimates would more accurately represent the actual values. Weighting was especially important when the area at high risk was relatively small compared with the size of the spatial filter. SMR was computed using the indirect standardization method. The equations were:
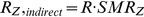
(1)

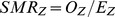
(2)

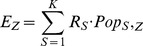
(3)Where 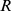 is the crude disease rate in the standard population, 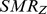 is the standardized morbidity rate at area 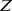, 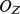 is the number of observed cases at area 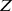, 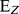 is the number of expected cases at area 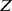, 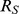 is the stratum-specific disease rate for age and gender stratum, 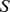, in the standard population, and 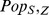 is the population size of age-gender stratum, 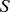, at area 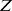.

### Scan statistics method

The scan statistics method is used to detect the clusters in a point process or to test whether a one-dimensional point process is purely random [36]. The spatial scan statistics method proposed by Kulldorff [25], [37], [38] was used to detect local clusters. A purely spatial analysis based on the Poisson model was used to explore the annual purely spatial clusters of HFMD incidence. The purely spatial scan statistic imposes a circular (or elliptical) window, which is centered on each geographical area throughout the study region. The radius of the window varies continuously in size according to the variations in population size in the area. The Poisson model assumes that the number of HFMD cases in each location follows a Poisson distribution. Under the null hypothesis, and when there are no covariates, the number of expected cases in each location is proportional to the population size. The Poisson model probability function is presented in Equation 4 [38], where 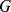 is the size of the entire study area, 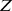 is a subzone of 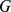, 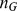 is the number of observed cases in 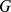,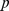 is the probability for observed cases inside 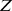,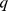 is the probability for observed cases outside 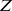, and 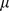 is the population size at risk. 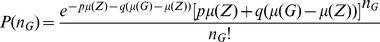
(4)Poisson approximation is especially useful when covariates can be included in the analysis [38]. The covariates age and gender were included in our analysis. The age-gender distribution of the HFMD cases and of the general population varied widely at the township level. Local clusters changed after adjusting for age and gender, which helped reveal hidden factors leading to local clusters. The number of expected cases was measured using the indirect standardization method presented above (Equation 3).

We used the Space-Time Permutation model for the early detection of disease outbreaks, which uses only numbers of cases to detect space-time clusters of HFMD occurrence[39]–[41]. This model is defined using a minimal number of assumptions about the time, geographical location, and size of an outbreak, and adjusts for natural purely spatial and purely temporal variation. It is an important tool and is used for early disease detection by local and national health department surveillance systems. The Space-Time Permutation model uses the probability function presented in Equation 5 [39], where 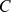 is the total number of observed cases, 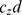 is the number of observed cases at area, 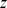, during day, 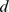,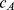 is the number of observed cases in a specific cylinder, 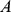, with a circular geographical base, and a height corresponding to time (the number of days). 
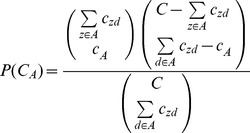
(5)The null hypothesis for the two probability models is that the rate (Poisson model) or the independence of the cases in space and time (Space-Time Permutation model) is the same within, and outside of, the scanning window. A likelihood ratio and the relative risk are calculated to test the hypothesis for each scanning window. The P-value for the detected clusters is evaluated using Monte Carlo simulation. The scanning window with the maximum likelihood is considered to be the most likely cluster. The other windows for which the likelihood value is statistically significant are defined as the secondary clusters, and are ranked according to their likelihood ratio test statistics. Scan statistics analyses were performed using SaTScan v9.1.1 software (Boston, MA, USA) [42]. ArcGIS V10.1 (ESRI, Redlands, CA, USA) was used to visualize the results of the scan statistical analysis.

## Results

### Epidemiological features

A total of 157,707 individual-level HFMD cases were reported in Beijing from January 2008 to December 2012. There were 1465 severe, and 33 fatal, cases. Twenty-three of the deaths occurred between the months of May and July. Most of the cases were in the 0 to 9-year age group (97.05%; 153,060 cases) (Table 1). Most of cases were preschoolers who were children that resided at home (52.32%) or in nurseries (41.56%). The remainder (6.13%) of the cases were students and adults. Among the 7,244 genotyped cases, CoxA16, EV71, and other EV accounted for 42.12, 42.37 and 15.52% of the infections, respectively. The predominant pathogens were CA16 (2009, 2011–1012) and EV71 (2008, 2010).

**Table 1:** Social-demographic characteristics of HFMD cases and the pathogen types of a subset of cases, Beijing, 2008–2012.

Group	2008	2009	2010	2011	2012	Total
**Age**						
0–4	14959(81.10)	20512(83.78)	36187(79.69)	24456(79.29)	28853(74.89)	124967(79.24)
5–9	2855(15.48)	3333(13.61)	7892(17.38)	5460(17.70)	8553(22.20)	28093(17.81)
10–14	411(2.23)	369(1.51)	736(1.62)	528(1.71)	619(1.61)	2663(1.69)
15+	221(1.20)	269(1.10)	593(1.31)	398(1.29)	503(1.31)	1984(1.26)
**Occupation**						
Nursery kids	7009(38.00)	9597(39.20)	19330(42.57)	13511(43.81)	16090(41.76)	65537(41.56)
Scattered kids	10080(54.65)	13552(55.35)	23472(51.69)	15562(50.46)	19840(51.50)	82506(52.32)
Other	1357(7.36)	1334(5.45)	2606(5.74)	1769(5.74)	2598(6.74)	9664(6.13)
Pathogen*						
CoxA16	76(16.14)	459(52.04)	626(30.37)	607(43.23)	1283(52.89)	3051(42.12)
EV71	347(73.67)	316(35.83)	988(47.94)	594(42.31)	824(33.97)	3069(42.37)
Other EV	48(10.19)	107(12.13)	447(21.69)	203(14.46)	319(13.15)	1124(15.52)
Missing data	17975	23601	43347	29438	36102	150463
**Gender**						
Male	11263(61.06)	14826(60.56)	27371(60.28)	18473(59.90)	23079(59.90)	95012(60.25)
Female	7183(38.94)	9657(39.44)	18037(39.72)	12369(40.10)	15449(40.10)	62695(39.75)
Gender ratio	1.57	1.54	1.52	1.49	1.49	1.52
**Total**	18446	24483	45408	30842	38528	157707

Figures in brackets refer to the percentage of total cases in the corresponding year and group. In Group “Pathogen*”, the figures in brackets refer to the percentage of the number of HFMD cases those were tested for the pathogen associated with the infection in the corresponding year and subgroup. “Missing data” refers to the cases without pathogen test results.

Among the total HFMD cases, 95,012 were male, and 62,695 were female. The average male-to-female sex ratio was 1.52 (1.57 in 2008, 1.54 in 2009, 1.52 in 2010, 1.49 in 2011, and 1.52 in 2012). Total HFMD incidence (1/100,000) was 164.3 during the study period (104.2 in 2008, 131.6 in 2009, 231.5 in 2010, 152.8 in 2011, 186.2 in 2012) (Table 2). HFMD incidence was highest in the 0 to 4 year age group. The proportion of this age group was the highest (83.78%) in 2009, and the lowest (74.89%) was in 2012. The proportion of HFMD cases in the 5 to 9 year age group increased from 13.61% in 2009 to 22.2% in 2012. Incidence in this age group increased from 632.3 in 2008 to 1,621.2 in 2012. The sex ratio of HFMD cases varied among the different age-gender groups (Table 3). For the 0 to 14-year age group, the male morbidity rate was usually higher than the female morbidity rate. However, for the 15 to 50+-year age groups, the female morbidity rate was slightly higher than the male morbidity rate. Generally speaking, the HFMD incidence for males was significantly higher than the incidence for females.

**Table 2:** Age-specific incidence (1/100,000) of reported HFMD cases, 2008–2012.

Age(y)	2008	2009	2010	2011	2012	Total
**0–4**	2,414.8(81.10)	3,152.8(83.78)	5,275.1(79.69)	3,463.0(79.29)	3,986.3(74.89)	650.8(79.24)
**5–9**	632.3(15.48)	702.9(13.61)	1,578.4(17.38)	1,060.7(17.70)	1,621.2(22.20)	146.3(17.81)
**10–14**	90.8(2.23)	77.7(1.51)	146.9(1.62)	102.4(1.71)	117.1(1.61)	13.9(1.69)
**15–19**	8.6(0.45)	8.5(0.35)	16.9(0.40)	10.2(0.37)	10.0(0.29)	3.0(0.37)
**20–24**	1.9(0.24)	1.9(0.19)	4.9(0.28)	2.4(0.21)	3.9(0.28)	2.0(0.25)
**25–29**	1.2(0.14)	2.2(0.20)	3.4(0.18)	2.7(0.22)	3.3(0.22)	1.6(0.19)
**30–34**	2.2(0.20)	2.6(0.18)	6.3(0.25)	4.3(0.26)	5.6(0.28)	2.0(0.24)
**35–39**	1.5(0.13)	1.7(0.11)	3.3(0.12)	2.5(0.14)	2.4(0.11)	1.0(0.12)
**40–44**	0.2(0.02)	0.4(0.03)	1.2(0.04)	0.9(0.05)	1.5(0.07)	0.4(0.05)
**45–49**	0.1(0.01)	0.3(0.02)	0.6(0.02)	0.3(0.02)	0.5(0.02)	0.1(0.02)
**50+**	0.1(0.02)	0.1(0.01)	0.1(0.01)	0.2(0.03)	0.2(0.03)	0.2(0.02)
**Total**	104.2(100)	131.6(100)	231.5(100)	152.8(100)	186.2(100)	164.3(100)

Figures in the brackets refer to the percentage of the total cases in the corresponding year and group.

**Table 3:** Age-gender incidence (1/100,000) of reported HFMD cases, 2008–2012.

Age(y)	2008		2009		2010		2011		2012		Total
	Male	Female	Male	Female	Male	Female	Male	Female	Male	Female	
**0–4**	2,824.3	1,959.9	3,641.9	2,609.4	6,083.4	4,377.2	3,956.5	2,914.7	4,564.0	3,344.6	650.8
	(81.75)	(80.08)	(84.10)	(83.29)	(80.23)	(78.87)	(79.60)	(78.84)	(75.32)	(74.24)	(79.24)
**5–9**	720.2	532.4	805.9	585.8	1,774.8	1,355.1	1,204.4	897.5	1,823.2	1,391.7	146.3
	(15.36)	(15.66)	(13.71)	(13.46)	(17.25)	(17.58)	(17.85)	(17.48)	(22.17)	(22.24)	(17.81)
**10–14**	96.4	84.7	81.0	74.0	159.7	132.8	109.7	94.3	127.2	105.9	13.9
	(2.03)	(2.53)	(1.36)	(1.73)	(1.53)	(1.75)	(1.61)	(1.87)	(1.53)	(1.72)	(1.69)
**15–19**	6.7	10.7	8.0	9.0	16.3	17.6	10.0	10.6	8.7	11.4	3.0
	(0.30)	(0.68)	(0.29)	(0.45)	(0.34)	(0.49)	(0.31)	(0.44)	(0.23)	(0.39)	(0.37)
**20–24**	1.8	1.9	1.5	2.2	3.3	6.6	1.4	3.5	3.0	4.9	2.0
	(0.20)	(0.31)	(0.13)	(0.28)	(0.16)	(0.46)	(0.10)	(0.36)	(0.19)	(0.43)	(0.25)
**25–29**	1.2	1.2	1.9	2.5	3.3	3.5	1.8	3.7	1.9	4.8	1.6
	(0.12)	(0.17)	(0.15)	(0.28)	(0.15)	(0.23)	(0.12)	(0.36)	(0.11)	(0.38)	(0.19)
**30–34**	1.7	2.7	1.7	3.6	5.5	7.1	4.0	4.7	5.4	5.8	2.0
	(0.13)	(0.29)	(0.10)	(0.31)	(0.19)	(0.34)	(0.21)	(0.34)	(0.23)	(0.34)	(0.24)
**35–39**	1.3	1.8	1.7	1.7	2.5	4.1	2.2	2.8	2.6	2.2	1.0
	(0.10)	(0.18)	(0.10)	(0.13)	(0.08)	(0.18)	(0.11)	(0.19)	(0.11)	(0.12)	(0.12)
**40–44**	0.1	0.3	0.2	0.7	1.1	1.3	1.1	0.6	1.9	1.1	0.4
	(0.01)	(0.03)	(0.01)	(0.05)	(0.04)	(0.06)	(0.05)	(0.04)	(0.08)	(0.06)	(0.05)
**45–49**	0.0	0.3	0.2	0.3	0.6	0.5	0.2	0.4	0.4	0.5	0.1
	(0.00)	(0.03)	(0.01)	(0.02)	(0.02)	(0.02)	(0.01)	(0.02)	(0.02)	(0.03)	(0.02)
**50+**	0.0	0.1	0.1	0.0	0.0	0.1	0.1	0.3	0.2	0.3	0.2
	(0.01)	(0.04)	(0.02)	(0.00)	(0.00)	(0.01)	(0.01)	(0.06)	(0.02)	(0.05)	(0.02)
**Total**	123.2	83.9	154.4	107.3	270.3	190.1	177.2	126.7	216.0	154.4	821.3
	(100)	(100)	(100)	(100)	(100)	(100)	(100)	(100)	(100)	(100)	(100)

Figures in the brackets refer to the percentage of the total cases in the corresponding year and group.

**Figure 2:**
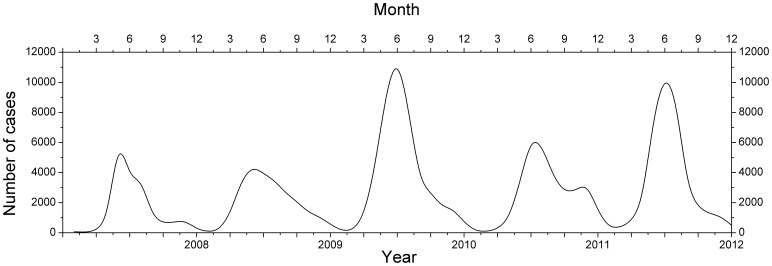
Monthly distribution of Beijing HFMD cases, 2008–2012.

The monthly distribution of HFMD cases exhibited significant seasonality and periodicity (Figure 2). The annual peaks in incidence mostly occurred between April and July. Incidence peaked twice in 2011, in the summer and in the autumn (between October and November) seasons. The annual number of HFMD cases was the lowest (18,446) in 2008 and the greatest (45,408) in 2010.

### LISA cluster maps

**Figure 3:**
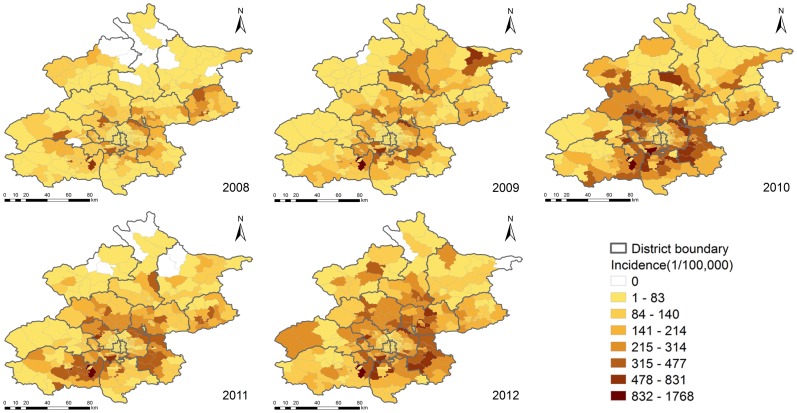
Annual HFMD incidence at the township level in Beijing, 2008–2012.

**Figure 4:**
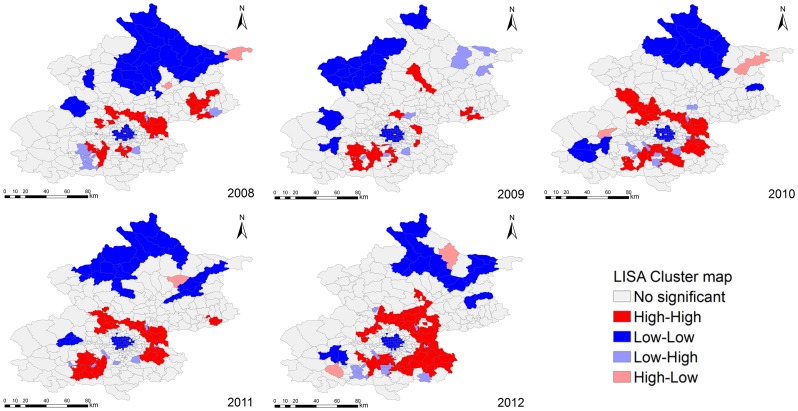
Univariate Local Indicators of Spatial Association (LISA) cluster maps for Beijing HFMD incidence, 2008–2012.

Figure 3 showed the distribution of HFMD incidence in Beijing at the township level. LISA analysis of HFMD incidence that occurred from 2008 to 2012 identified different patterns of spatial association for HFMD epidemics (Figure 4). The High-High and Low-Low townships suggested the clustering of similar values for HFMD incidence, whereas the Low-High and High-Low townships indicated spatial outliers. It could be seen that the urban-rural transition zones around the old city in Beijing showed the strengthened High-High positive spatial association for HFMD incidence year by year, the old city of Beijing showed the stable Low-Low positive spatial association for HFMD incidence. The townships showing negative spatial association were mainly scattered in south and northeast Beijing.

### Relative risk

**Figure 5:**
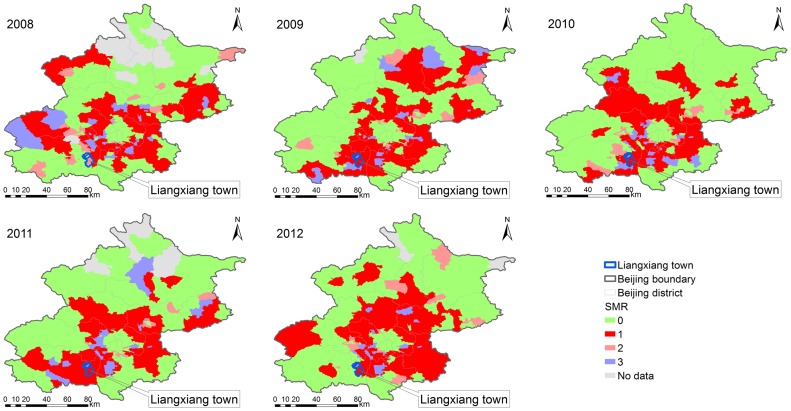
Township level annual standardized morbidity rate (SMR), by spatial filtering and scan statistics methods, 2008–2012. ‘0’ represents the areas with an SMR<1 simultaneously identified as cold spots by the two methods, ‘1’ represents the areas with an SMR>1 simultaneously identified as hot spots by the two methods, ‘2’ represents the areas with an SMR>1 identified as hot spots by scan statistics method but with an SMR<1 as cold spots by spatial filtering method, ‘3’ represents the areas with an SMR>1 identified as hot spots by spatial filtering method but with an SMR<1 as cold spots by scan statistics method, ‘No data’ represents the areas without the occurrence of HFMD cases.

The relative risk of HFMD evaluated by estimating the SMR. Areas with an SMR>1 were identified as hot spots, and areas with an SMR<1 were considered to be cold spots. The spatial filtering and scan statistics revealed the geographic patterns in relative risk (Figure 5). Similar to the results of a previous study [13], the areas at the highest relative risk were located mainly in the annulus transition zones between the old city and the outlying districts of Beijing, in which the percentage of the population at risk was 35.66% in 2008, 36.34% in 2009, 36.87% in 2010, 33.89% in 2011 and 39.58% in 2012. Liangxiang town in the Fangshan district was a hotspot with a significantly higher relative risk (ranged from 5.82 in 2012 to 12.58 in 2009 by scan statistics method, ranged from 5.13 in 2012 to 10.98 in 2009 by spatial filtering method). The differences in outcome between the two methods is because the spatial filtering method can be used to estimate the relative risk for the areas with zero HFMD cases and produce an isarithmic map of disease risk. The scan statistics method does not provide an estimate of relative risk unless cases occur in the area in question.

### Spatial Clusters

**Figure 6:**
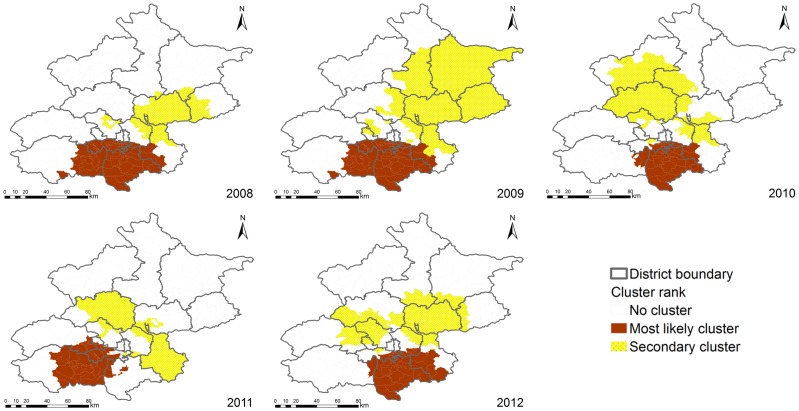
The detected purely spatial clusters of Beijing HFMD, 2008–2012.

A total of 154,463 cases matched to the centers of the townships (97.94% of the total reported cases). Figure 6 presents the statistically significant spatial clusters (including the most likely cluster and several secondary clusters) that were detected by the purely spatial scan statistics analysis based on the Poisson model. The locations and sizes of the spatial clusters varied from year to year. During the epidemic periods, the most likely clusters occurred mostly in the Daxing district (2008–2010, 2012), the east Fangshan district (2008, 2009, 2011), the southwest Tongzhou district (2008, 2009, 2010, 2012), and the Fengtai district (2008, 2009). Table 4 includes the locations, sizes, relative risks, and P-values of the most likely spatial clusters, and indicates the Beijing HFMD hotspots for the 2008–2012 study period.

**Table 4:** The detected most likely purely spatial clusters of Beijing HFMD, 2008–2012.

Year	Center	Cluster areas (n)	Radius (km)	Observed cases	Expected cases	Relative risk	P-value
2008	Yufa town, Daxing	56	41.65	6185	4474.22	1.58	0.001
2009	Yufa town, Daxing	56	41.65	9323	5895.43	1.95	0.001
2010	Weishanzhuang town, Daxing	36	24.65	11641	7444.33	1.76	0.001
2011	Xinzhen community, Fangshan	40	24.00	6243	3840.32	1.79	0.001
2012	Anding town, Daxing	39	28.76	9787	6361.95	1.73	0.001

### Space-time Clusters

**Figure 7:**
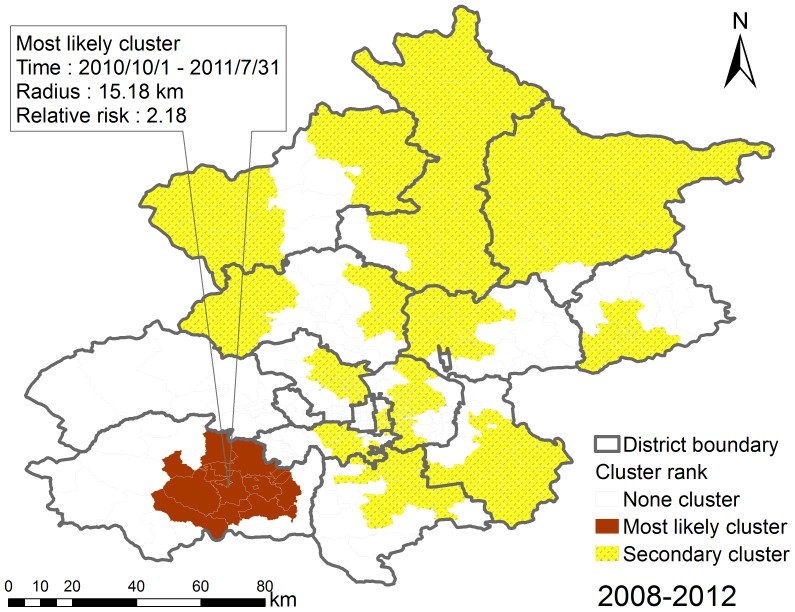
The detected space-time clusters of Beijing HFMD, 2008–2012.

We detected the statistically significant space-time clusters using space-time scan statistics that were based on the Space-Time Permutation model. Fourteen space-time clusters were detected, including one most likely cluster and thirteen secondary clusters, indicating that the occurrence of Beijing HFMD displayed space-time heterogeneity (Figure 7 and Table 5). The most likely cluster (relative risk was 2.18, P-value was 0.001) was located in the mid-east part of the Fangshan district, southwest of Beijing during the period from 2010/10/1 to 2011/7/31, including 16 townships. The secondary clusters were scattered in the different Beijing districts, with relative risk ranging from 1.23 (from 2010/10/1 to 2012/12/31, P-value was 0.001) to 3.7 (from 2009/9/1 to 2009/12/31, P-value was 0.001), including 147 townships.

**Table 5:** The detected significant space-time clusters of Beijing HFMD, 2008–2012.

Cluster	Cluster areas(n)	Radius(km)	Start time	End time	Relative risk	P-value
1	16	15.18	2010/10/1	2011/7/31	2.18	0.001
2	30	44.53	2009/3/1	2009/9/30	2.11	0.001
3	5	4.70	2008/7/1	2010/5/31	1.35	0.001
4	9	12.81	2009/9/1	2009/12/31	3.70	0.001
5	1	0.00	2008/5/1	2008/6/30	2.75	0.001
6	19	15.30	2012/6/1	2012/11/30	1.47	0.001
7	15	5.59	2008/6/1	2008/11/30	1.68	0.001
8	15	12.63	2010/3/1	2010/6/30	1.30	0.001
9	10	28.00	2010/6/1	2010/10/31	1.79	0.001
10	6	17.10	2011/9/1	2012/8/31	1.40	0.001
11	3	2.18	2010/9/1	2010/11/30	3.20	0.001
12	23	5.39	2010/10/1	2012/12/31	1.23	0.001
13	9	5.21	2011/11/1	2012/1/31	2.02	0.001
14	2	3.39	2009/6/1	2009/7/31	2.21	0.001

Notes: ‘1’ represents ‘Most likely cluster’; ‘2–14’ represent 13 ‘Secondary clusters’.

## Discussion

HFMD epidemics varied greatly over time and age-gender groups. Male children, especially in the 0 to 4-year age group, were more susceptible to HFMD and should be considered to be a group at significant risk. There were two peaks in HFMD occurrence during the warm season (between April and July) and the cold season (between October and November). LISA analysis revealed that the circle areas with High-High positive spatial association for HFMD incidence were located around the old city, Low-Low positive spatial association for HFMD incidence significantly occurred in north Beijing, and the local clusters of negative spatial association wandered in south and northeast Beijing. Spatial filtering combined with scan statistics methods could provide verification, and more details, about the hotspots with the high relative risks so that more timely and precise prevention and control of disease outbreaks could be implemented. These two methods have been widely used to detect hot spots and cold spots for infectious disease epidemics and may serve as a useful adjunct to disease surveillance, particularly in areas with limited resources. The cluster detection results from both methods indicated that the Beijing HFMD epidemics propagated in a composite space-time domain rather than exhibiting purely spatial or purely temporal variation. The annulus transition zones around the old city were revealed by the two methods to be at significantly higher relative risk, and southwest (the Fangshan and Daxing districts) and southeast (the Tongzhou district) Beijing displayed increasing relative risk during the study period. These areas should receive particular attention during future public health planning and resource allocation.

We found that the scan statistics method could detect HFMD clusters over space and time and reveal multi-dimensional information about the patterns of the epidemics. However, the spatial scan statistics method includes the assumption of circular or cylinder scanning windows to detect the clusters, which may not represent the actual shapes of the clusters. Because of this limitation, we plan to explore other methods for future spatial epidemiological analyses. The spatial filtering method produced a useful isarithmic map of relative risk, but scan statistics method could not estimate the relative risk of HFMD epidemics for the areas without HFMD cases. Therefore, our approach (i.e., combining the two popular cluster detection methods) allowed us to rigorously and comprehensively identify and describe the space-time patterns of individual-level HFMD cases throughout a long-term period.

Other limitations of this study deserve mention. First, only 4.59% (7,244 cases in all) of the reported HFMD cases for the years from 2008 to 2012 were tested for the pathogen associated with the infection, which may have reduced the power of the virological surveillance data analysis. Second, missing spatial location information for the cases may have, to some extent, reduced the accuracy of the results.

In summary, the analysis of multi-dimensional variables (e.g., age, gender, time, and space) and the detection of clusters of disease epidemics could support surveillance systems by identifying the most likely hotspots for disease control and prevention, earlier and with greater precision. The variations in HFMD epidemics over population, space, and time that were revealed by this study emphasize the need for more thorough research about the driving forces and risk factors (climate, geography, environment, and social-economic) that contribute to outbreaks of HFMD.
